# Incidence of clinical malaria, acute respiratory illness, and diarrhoea in children in southern Malawi: a prospective cohort study

**DOI:** 10.1186/s12936-021-04013-5

**Published:** 2021-12-20

**Authors:** Tinashe A. Tizifa, Alinune N. Kabaghe, Robert S. McCann, William Nkhono, Spencer Mtengula, Willem Takken, Kamija S. Phiri, Michele van Vugt

**Affiliations:** 1 Center for Tropical Medicine and Travel Medicine, Department of Infectious Diseases, Division of Internal Medicine, University of Amsterdam, Amsterdam University Medical Center, Location Academic Medical Center, Amsterdam, The Netherlands; 2 School of Public Health and Family Medicine, College of Medicine, University of Malawi, Blantyre, Malawi; 3 Laboratory of Entomology, Wageningen University & Research, Wageningen, The Netherlands; 4 Center for Vaccine Development and Global Health, University of Maryland School of Medicine, Baltimore, USA

**Keywords:** Malaria, Incidence, Acute respiratory infections, Diarrhoea, Under-five children, Community engagement, Malawi

## Abstract

**Background:**

Malaria, acute respiratory infections (ARIs) and diarrhoea are the leading causes of morbidity and mortality among children under 5 years old. Estimates of the malaria incidence are available from a previous study conducted in southern Malawi in the absence of community-led malaria control strategies; however, the incidence of the other diseases is lacking, owing to understudying and competing disease priorities. Extensive malaria control measures through a community participation strategy were implemented in Chikwawa, southern Malawi from May 2016 to reduce parasite prevalence and incidence. This study assessed the incidence of clinical malaria, ARIs and acute diarrhoea among under-five children in a rural community involved in malaria control through community participation.

**Methods:**

A prospective cohort study was conducted from September 2017 to May 2019 in Chikwawa district, southern Malawi. Children aged 6–48 months were recruited from a series of repeated cross-sectional household surveys. Recruited children were followed up two-monthly for 1 year to record details of any clinic visits to designated health facilities. Incidence of clinical malaria, ARIs and diarrhoea per child-years at risk was estimated, compared between age groups, area of residence and time.

**Results:**

A total of 274 out of 281 children recruited children had complete results and contributed 235.7 child-years. Malaria incidence was 0.5 (95% CI (0.4, 0.5)) cases per child-years at risk, (0.04 in 6.0–11.9 month-olds, 0.5 in 12.0–23.9 month-olds, 0.6 in 24.0–59.9 month-olds). Incidences of ARIs and diarrhoea were 0.3 (95% CI (0.2, 0.3)), (0.1 in 6.0–11.9 month-olds, 0.4 in 12.0–23.9 month-olds, 0.3 in 24.0–59.9 month-olds), and 0.2 (95% CI (0.2, 0.3)), (0.1 in 6.0–11.9 month-olds, 0.3 in 12.0–23.9 month-olds, 0.2 in 24.0–59.9 month-olds) cases per child-years at risk, respectively. There were temporal variations of malaria and ARI incidence and an overall decrease over time.

**Conclusion:**

In comparison to previous studies, there was a lower incidence of clinical malaria in Chikwawa. The incidence of ARIs and diarrhoea were also low and decreased over time. The results are promising because they highlight the importance of community participation and the integration of malaria prevention strategies in contributing to disease burden reduction.

**Supplementary Information:**

The online version contains supplementary material available at 10.1186/s12936-021-04013-5.

## Background

Worldwide, the three most common causes of death of under-five children are pneumonia, diarrhoea and malaria [1]. In sub-Saharan Africa, an estimated 2.5 million children under-five years old die each year from one of these three preventable and treatable diseases [2]. High population density, poor housing and sanitation infrastructure, inadequate knowledge, malnutrition, and poor access to quality health, education, and employment all contribute to the risk of pneumonia, diarrhoea and malaria in this region [3]. Addressing these contributing factors may reduce the occurrence and therefore, morbidity and mortality associated with these diseases in rural communities. Reducing morbidity and mortality from infectious diseases has potential economic benefits: there is no income loss due to treatment and death costs, and there is an increase in productivity because healthy people are better able to participate in economic activities that will improve their lives [4, 5]. In addition, scaling up interventions such as vaccines (pneumococcal and rotavirus), insecticide-treated mosquito nets (ITNs), structural improvements of houses, and nutritional supplementation may further reduce the incidence of these diseases [6–9].

Uptake and effectiveness of existing efficacious health interventions, in all communities, requires community buy-in. To improve the uptake and effectiveness of health interventions and promote positive health behaviour in communities, several strategies have been used in the past. These strategies include active community engagement and participation, information, education, and communication (IEC), communication for behavioural-impact (COMBI), behaviour change communication (BCC), and school-based health education [10–15]. In any setting, health promotion and community engagement strategies should consider factors such as culture, pre-existing beliefs, community structures, literacy levels and education, communication language, transportation options, and availability of resources for personnel support and effective programme operation [16].

A community participation strategy was utilized to increase awareness and participation in malaria prevention and control in Chikwawa, Southern Malawi [17]. This community participation strategy included activities such as leadership training for local volunteers, known as health animators, who were tasked with conducting IEC on malaria and involving the local community in the implementation of malaria control strategies using existing community structures. The goal of this community-based approach was to influence a change in mind-set of the entire community to promote self-reliance through understanding malaria and its control [18, 19]. The approach was led and integrated into the community by building capacity within the communities in malaria prevention and control using regular community malaria workshops and sensitization [17]. Complementary malaria control strategies, such as house improvement (HI) and larval source management (LSM), were introduced in the community in addition to the government-recommended ITNs to achieve a significant reduction in malaria burden [20].

This study aimed to determine the trend of incidence of clinical malaria, acute respiratory infections (ARIs), and acute diarrhoea in under-five children residing in rural communities where a community-based malaria prevention strategy, using HI and LSM, was implemented in addition to government-recommended ITNs. The study also identified risk factors associated with clinical malaria, ARI and diarrhoea. Investigation of these conditions within the under-five population in a rural setting could provide a more holistic approach to the treatment and control of all conditions, especially in sites that are remote and hard to reach.

## Methods

### Study design

This was a prospective cohort study of children aged 6–48 months. Study participants were recruited from households sampled in a rolling malaria indicator survey (rMIS) [21, 22].

### Study site and setting

The study was implemented in a rural community surrounding the Majete Wildlife Reserve (MWR) in Chikwawa, southern Malawi, from September 2017 to May 2019. The study area was within the catchment of the Majete Malaria Project (MMP), a community-based malaria control project (Additional file 1: Fig. S1). The surveys were carried out in 65 villages with a total population of about 25,000 people and 6600 households [21]. The health facilities serving the area consist of one district referral hospital and 26 primary healthcare facilities (village clinics and health centres). Some of the services offered at these settings include immunization, under-five clinics, maternity, and outpatient department. The district hospital offers secondary healthcare, requiring hospital admission. This study was conducted in three main sites which were referred to as focal areas A, B and C, representing hubs for community development.

### Sample size and sampling technique

A sample size of 285 children was calculated using the statistical z-test with the following formula N = (z_1-α/2_/ε)^2^ to calculate the sample size of the cohort, where z_1-α/2_ is the standard deviation for the probability *p* and ε is the relative precision [23]. The confidence level was set at 90%, relative precision was 10%; the addition of 5% of the total was done to account for attrition.

All children in six rMIS [21] household sampling rounds aged 6–48 months were eligible. Every two months a sample of 270 households (90 per focal area) were sampled using inhibitory spatial random sampling [24]. These households were selected as described in [19, 25] from a demographic database covering the research area, and surveys were conducted in these households over a 6 to 8-week period in each round with short breaks between rounds (5 to 15 days) [19, 25]. Surveys were conducted simultaneously in each focal area. In brief, rMIS involved small teams of study staff containing a nurse with at least 2–4 research assistants visiting sampled households [22].

### Data collection

The study team used an electronic structured questionnaire in a mobile-based application called the Open Data Kit (ODK).

#### Training

The study team consisted of 12 research assistants and three study nurses. The study team underwent a two-day protocol training including identifying sampled households, administering consent, and completing the questionnaire using sick-visit cards. Research assistants booked guardians for recruitment and follow-up and nurses administered the questionnaire, recorded sick-visit cards details, and conducted clinical assessments.

Health workers from the health facilities within the study site involved in the clinical management of under-five children underwent a one-day orientation of the study procedures. These health workers were trained on how to record the malaria diagnosis and malaria rapid diagnostic test (RDT) results on the sick-visit cards provided to participants. A research nurse recorded details from the health passport to the sick-visit card if the health worker did not record the sick-visit in the sick-visit card; health workers are required to record details of each clinical consultation in a health passport as part of their routine work.

#### Recruitment

At the recruitment visit, a questionnaire was administered to a consenting head of the household or the child's guardian. The age and gender of the study participant was recorded. Data on the features of the house, including the main materials of the walls, roof and floor, and the presence of eaves were obtained and validated. In addition, ownership of ITNs and household items were recorded. A clinical assessment, including temperature and anthropometric measurements, was conducted. The participants were tested for malaria using a RDT (SD Bioline malaria Ag Pf HRP-2 Standard Diagnostics Inc, Gyeonggi-do, Republic of Korea) and haemoglobin (Hb) level was measured using Hemocue 301 (Haemocue, Angelholm, Sweden) machines in children with signs and symptoms suggestive of malaria. Children with uncomplicated malaria were prescribed first-line treatment of artemether-lumefantrine (AL), while those with Hb levels less than 11 g/dl were referred to the nearest health facility for care. Recruited participants were given a sick-visit card which was pinned to their health passports. Health workers are expected to record the details of each clinical consultation in a health passport as part of their routine work.

#### Follow-up

Study personnel visited the participants' households at two-monthly periods for 12 months to collect and replace sick-visit cards. The follow-ups conducted at months 2, 4, 8, and 10 involved the collection and replacement of sick-visit cards. However, malaria tests were conducted using RDT to children that had malaria symptoms during these visits. All children were screened for malaria symptoms at 6 and 12-month follow-up household visits, and only symptomatic children were tested for malaria using RDT. Malaria treatment was given to participants who tested positive for the disease, and the information was recorded on the sick-visit card as a clinical malaria case. Clinical events documented by health workers at health facilities as a result of acute diarrhoea and ARIs were transcribed from the sick-visit card by research nurses. After cross-referencing the data on the sick-visit card with the data on the health passport, the data were entered electronically into a tablet and sent to a remote server via an internet connection.

### Data sources and variables

*Clinical malaria* was defined according to the national malaria control programme revised guidelines for the treatment of malaria in Malawi [26] as signs and symptoms suggestive of malaria and positive RDT result during a sick-visit or at 6 or 12-month study visit. Symptoms of malaria listed in the revised guidelines for standard malaria treatment include fever, vomiting, headache, and malaise [26]. Only the first clinical malaria diagnosis was included for children who had a clinical malaria diagnosis more than once within 14 days.

*Acute diarrhoea* was defined according to the World Health Organization (WHO) as a clinical syndrome with acute onset of three or more loose or liquid stools in 24 h with the duration lasting several hours or days [27].

#### Acute respiratory infections

ARIs, classified as upper respiratory tract infections (URTIs) and lower respiratory tract infections (LRIs), are clinical syndromes involving the upper and lower airways. ARIs have also been defined according to WHO. URTIs are defined as a clinical syndrome characterized by the sudden onset of fever and cough or sore throat. For LRIs, pneumonia was the condition of interest. Pneumonia was defined as a clinical syndrome with at least one of the following: fever or cough, shortness of breath, and chest pains with appropriate antibacterial therapy initiated or recommended [28].

Three HOBO weather stations (Onset Computer Corporation, MA, USA), one in each focal area, measured hourly rainfall in millimetres (mm), the temperature in degrees Celsius (°C), and relative humidity as a percentage. Monthly average temperature and relative humidity together with the total rainfall were calculated from the weather data. Monthly averages of weather components (parts) from the three weather stations were used to calculate the overall weather for each month. This was used to compare to monthly incidence data.

### Statistical analysis

R version 4.0.2 was used to analyse data. For the overall incidence rate of clinical malaria, the total follow-up time was calculated from the total time in years between recruitment and study exit, whether this was at the end of the one year, lost to follow-up, relocation, or withdrawal. To account for the time, the children were not at risk of clinical malaria, 14 days were subtracted from the child-years follow-up with each case of clinical malaria which occurred. Univariate analysis for continuous variables such as age was conducted. Furthermore, reporting of median and inter-quartile range (IQR), and proportions for categorical variables was done. Principal component analysis (PCA) was used to create a wealth index from ownership of livestock (cattle, goats, sheep, chicken, etc.) and other assets (mobile phones, radios, television, bed, bicycle, toilet type, sofa, etc.). Each asset was assigned a score factor depending on the standard deviation (i.e., an asset which all households own or which no households own would have a minimum score). The incidence rate was calculated by dividing the total number of clinical outcomes by the time at risk. Incidence rates by a priori variables such as age, focal area and intervention arm are reported and compared. Comparison of the rates is reported as an incidence rate ratio (IRR) calculated as a ratio of the incidence rate in a particular group divided by the incidence rate in a comparison or reference group.

### Ethical considerations

The College of Medicine Research and Ethics Committee (COMREC) reviewed and approved the protocol (Certificate numbers P.11/14/1658). The study conformed to the principles of human subjects’ protection. Before the data collection, communities were sensitized and informed of the purpose of the study. The district health office and all health facilities were engaged and supported the implementation of the study. Consent was obtained from the parents and legal guardians of the children.

## Results

**Fig. 1:**
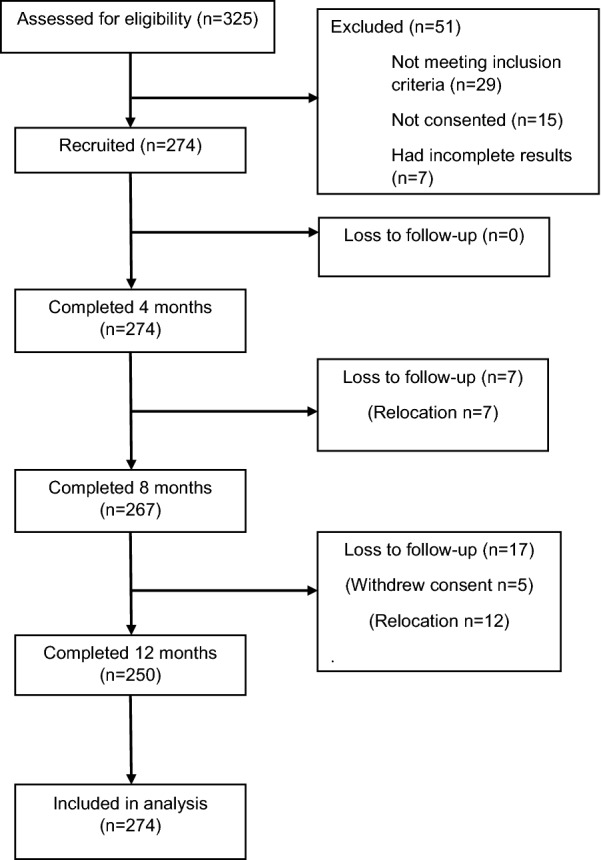
Flow-diagram of participants

A total of 281 children were recruited for the study out of 325 children who were from sampled households. There were 29 children aged > 48 months and 15 guardians refused consent. Seven of the 281 children had incomplete data and were excluded from the analysis; the remaining 274 children from 254 households had complete data and were included (Fig. 1); 250 (91.2%) children completed all the 12 months of follow-up. Overall, participants who were lost to follow-up were 24 (8.8%), some of whom had withdrawn consent and others had relocated out of the study catchment area.

The median age of the children was 25 months with 50.7% of the children aged between 24 and 48 months (IQR 16.0–35.0); 52.6% were female (Table 1). The mean Hb was 11.0 g/dl and 47.7% of the children had Hb above 11.0 g/dl. There was a high prevalence of stunting (33.5%). A total of 12 children tested positive for malaria by RDT at recruitment; 58.7% of households reported owning at least one ITN for sleeping.

**Table 1:** Demographic and health characteristics of the population at recruitment

Characteristic at recruitment	Children (n = 274)
Age in months (Median, IQR)	25 (16.0–35.0)
Gender: Female (%)	144 (52.6%)
Age categories in months	
6.0–11.9 months, n (%)	50 (18.2%)
12.0–23.9 months, n (%)	85 (31.0%)
24.0–48.0 months, n (%)	139 (50.7%)
Recruitment by focal area	
Focal Area A, n (%)	105 (38.3%)
Focal Area B, n (%)	78 (28.5%)
Focal Area C, n (%)	91 (33.2%)
Number of children by intervention arm	
Control, n (%)	82 (29.9%)
HI, n (%)	61 (22.3%)
LSM, n (%)	82 (29.9%)
HI and LSM, n (%)	49 (17.9%)
Anthropometry at recruitment	
HAZ < − 2 SD, n (%)	91 (33.5%)
WAZ < − 2 SD, n (%)	49 (18.0%)
WHZ < − 2 SD, n (%)	39 (14.3%)
RDT – positive at recruitment: n (%)	12 (4.4%)
Haemoglobin level in g/dl: mean SD	11.0 g/dl, 1.1
Anaemia classification, n (%)	
Normal Hb ≥ 11.0 g/dl	83 (47.7%)
Mild Hb 10–10.9 g/dl	66 (37.9%)
Moderate Hb 7.0–9.9 g/dl	24 (13.8%)
Severe Hb < 7.0 g/dl	1 (0.6%)
Socioeconomic status	
Wealth Score^a^: mean, SD	− 0.1, ± 2.0
Households owning ITN for sleeping	91 (58.7%)
Households with at least two ITNs (%)	36 (48.9%)
Presence of open eaves on house (%)	45 (29.0%)

IQR: Interquartile range; Hb: Haemoglobin; SD: Standard deviation; HAZ: Height for age z-score; WAZ: Weight for age z-score; WHZ: Weight for height z-score; HI: House improvement; LSM: Larval source management

^a^A composite measure of a household's cumulative living standard. Each household assigned a standardized score for each asset (bicycle, radio, TV, domestic animals)

### Clinical malaria incidence across the area of residence, intervention arm and age groups

There was a total of 110 malaria cases recorded from the 274 participants through passive case detection. The total duration of follow-up was 235.7 child-years, of which 231.5 were child-years at risk (Additional file 1: S1). The overall incidence of clinical malaria was 0.5 cases per child-years at risk (Table 2). The incidence of clinical malaria was highest in focal area B and across the HI and LSM intervention arm. The incidence of clinical malaria was highest in children aged 24.0–59.9 months at 0.6 cases per child-years at risk. Out of 274 children, the proportion of children with at least one clinical malaria case was 35.0%.

**Table 2:** Number of clinical malaria cases, incidence rates and incidence rate ratios (IRR) of the follow-up study

	Total child-years in the study	Total child-years at risk^a^	Children with at least one clinical malaria case: n (%)	Clinical malaria cases	Incidence rate: per child-years at risk (95% CI)	IRR (95% CI)
Overall	235.7	231.5	96 (35.0%)	110	0.5 (0.4–0.5)	–
Focal area						
A	105.0	103.7	29 (27.6%)	34	0.3 (0.2–0.4)	Reference
B	57.6	55.5	53 (67.9%)	56	1.0	3.3 (1.2–5.1)
C	73.0	72.2	14 (15.2%)	20	0.3 (0.2–0.4)	1.0 (0.6–1.7)
Intervention arm						
Control	69.5	68.5	21 (25.6%)	26	0.4 (0.3–0.5)	Reference
HI	58.6	58.3	5 (8.2%)	7	0.1 (0.1–0.2)	0.3 (0.1–0.7)
LSM	67.1	65.4	43 (52.4%)	44	0.7 (0.6–0.8)	1.8 (1.1–2.9)
HI & LSM	40.4	39.1	27 (55.1%)	33	0.8 (0.7–1.0)	2.0 (1.2–3.3)
Age in months						
6.0–11.9	44.9	44.8	2 (4.0%)	2	0.04 (0.01–0.2)	Reference
12.0–23.9	77.9	76.3	33 (38.8%)	41	0.5 (0.4–0.7)	12.5 (3.0–51.7)
24.0–59.9	112.8	110.2	61 (43.9%)	67	0.6 (0.5–0.7)	15.0 (3.7–61.2)

Reference for all calculations including IRR is from Rothman et al. [29]

The reference or comparison area/intervention arm/age-group have been labelled as Reference

^a^Calculated from subtracting 14 days from total child-years for each malaria case; child-years at risk also includes the period of follow-up of children who did not complete 12 months

### Temporal changes in climatic conditions and malaria incidence

**Fig. 2:**
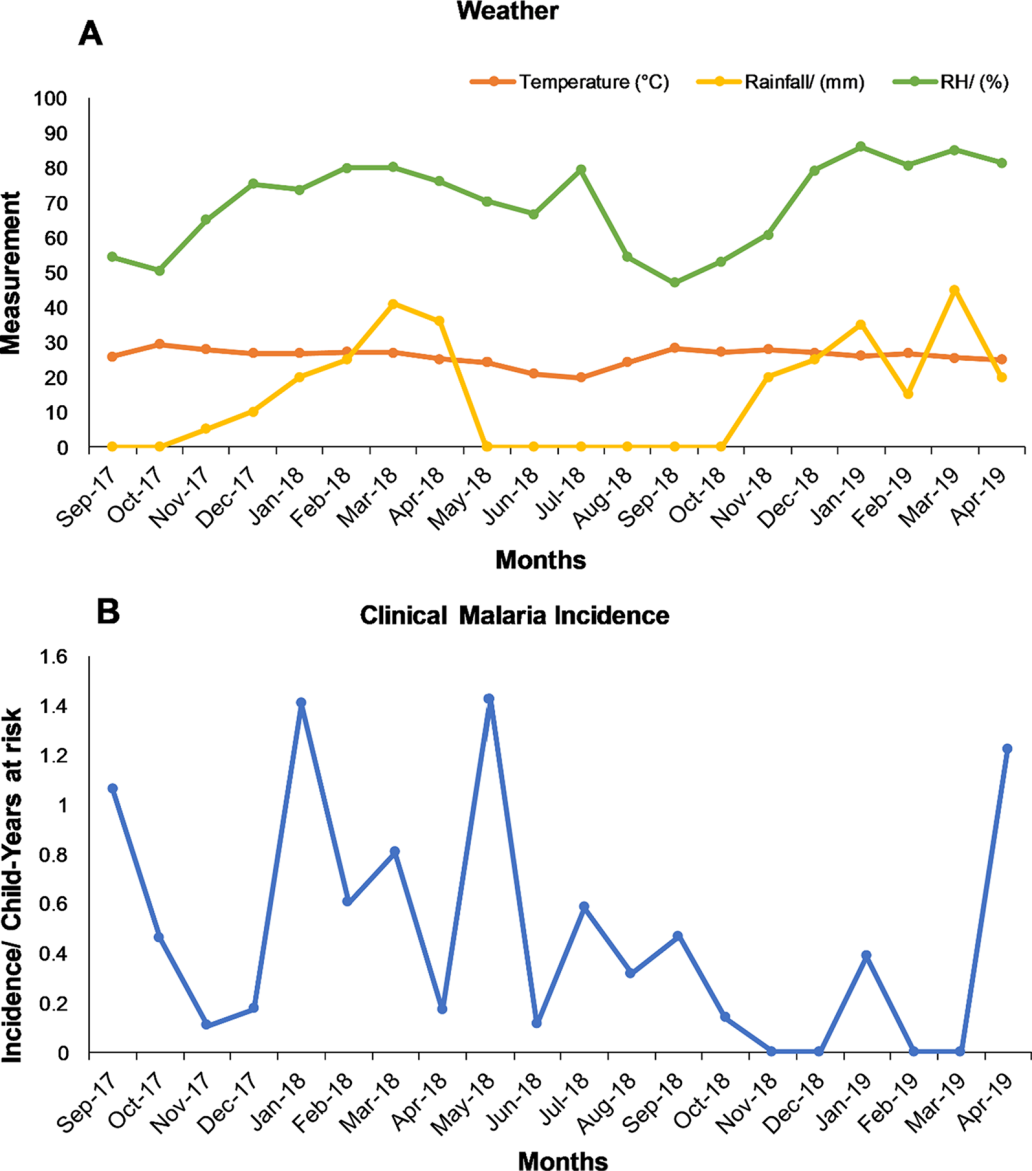
Monthly mean temperature, rainfall and relative humidity (**A**) and malaria (**B**)

Malawi has three annual seasons, namely hot-wet, cold-dry, and hot-dry seasons. In this study, there was no significant difference in the weather pattern across the focal areas. Figure 2 shows the pattern of malaria within the study period from September 2017 to April 2019. The hot-wet season ranges from November to April, cold-dry season from May to July, and hot-dry season from August to October. As shown in Fig. 2, there was an initial rise in malaria incidence in December 2017 (0.18 clinical malaria cases/child-years at risk) with the first peak arriving in January 2018 (1.4 clinical malaria cases/child-years at risk). This was followed by a decline in February followed by a small rise in March (0.8 clinical malaria cases/child-years at risk). The highest peak was reached in May 2018 (1.4 clinical malaria cases/child-years at risk) and this was followed by a drop in incidence with the lowest value being reached in November 2018 (0 clinical malaria cases/child-years at risk). A minor peak occurred a year later in January 2019 (0.3 clinical malaria cases/child-years at risk). The malaria peaks immediately follow the peaks in rainfall and relative humidity (RH). The mean monthly temperature varied between 20 and 30 °C.

### Hospital admissions and deaths

There were two hospital admissions due to severe malaria for the entire study period representing an incidence rate of 0.01 cases per child-years at risk. No hospitalizations occurred due to other conditions. No deaths were recorded during this period.

### Incidence of ARI and diarrhoea

A total of 47 acute diarrhoea cases were recorded over 235.7 child-years followed-up (Table 3). The overall incidence of diarrhoea was 0.2 cases per child-years at risk and was highest among those aged 12.0–23.9 months. There were 66 cases of URTI, with the overall incidence being 0.3 cases per child-years. Incidence for pneumonia was 0.3 cases per child-years, with 65 cases observed and the 12.0–23.9 and 24.0–59.0 months’ age groups having the highest risk.

**Table 3:** Incidence of ARIs and diarrhoea according to age groups

Condition	Age groups in months	Cases	Total child-years at risk	Incidence rate: cases per child-years at risk (95% CI)	IRR (95% CI)
Diarrhoea	6.0–59.0	47	235.7	0.2 (0.2–0.3)	
	6.0–11.9	5	44.9	0.1 (0.05–0.3)	Reference
	12.0–23.9	22	77.9	0.3 (0.2–0.4)	3.0 (1.1–7.9)
	24.0–59.0	20	112.8	0.2 (0.1–0.3)	2.0 (0.8–5.3)
Upper Respiratory Tract Infection (URTI)	6.0–59.0	66	235.7	0.3 (0.2–0.3)	
	6.0–11.9	5	44.9	0.1 (0.05–0.3)	Reference
	12.0–23.9	25	77.9	0.3 (0.2–0.4)	3.0 (1.2–7.8)
	24.0–59.0	36	112.8	0.3 (0.2–0.4)	3.0 (1.2–7.6)
Pneumonia	6.0–59.0	65	235.7	0.3 (0.2–0.3)	
	6.0–11.9	5	44.9	0.1 (0.05–0.3)	Reference
	12.0–23.9	28	77.9	0.4 (0.3–0.5)	4.0 (1.5–10.4)
	24.0–59.0	32	112.8	0.3 (0.2–0.4)	3.0 (1.2–7.7)

### Temporal changes in ARI and diarrhoea incidence

**Fig. 3:**
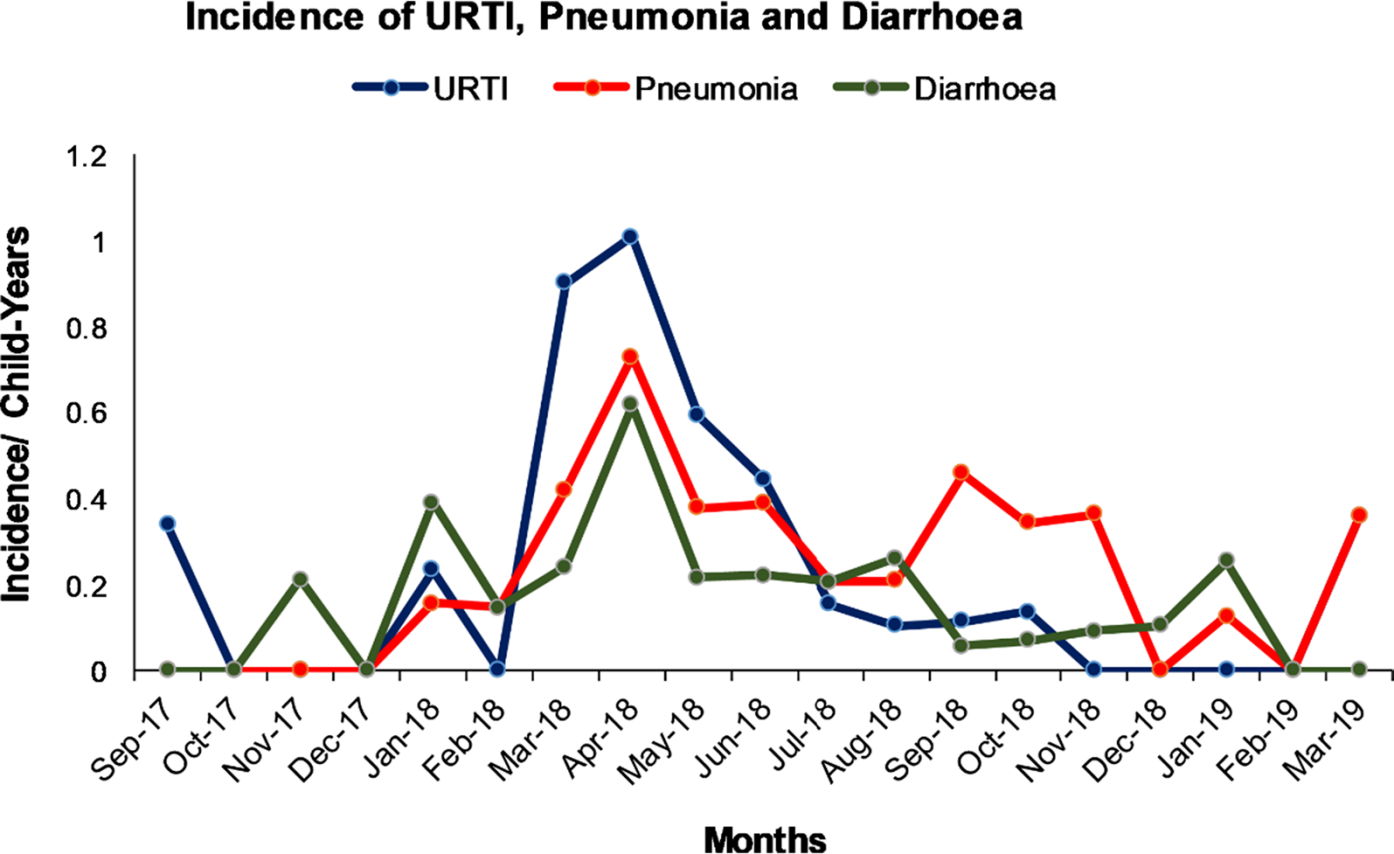
Temporal changes in ARI and diarrhoea incidence

Figure 3 shows the monthly variation of incidence of ARIs and diarrhoea. The first rise of incidence of URTI and diarrhoea was observed in January 2018 which then subsided and started rising in March 2018. The second and highest peak in incidence for URTI, pneumonia and diarrhoea was in April 2018. A partial rise of pneumonia was also observed in September 2018 which declined remarkably. Peaks in pneumonia and URTI immediately follow the drops in temperatures.

## Discussion

In this prospective cohort study in a rural area with a community engagement initiative on malaria prevention, overall incidence rates of clinical malaria, ARIs and diarrhoea were found to be fewer than 1 case per child-years at risk. There were significant temporal variations in disease incidence of all the diseases. The results of temporal measurements show when cases of malaria, ARIs and diarrhoea occurred. Previous estimates of malaria incidence within the same area are available, however no previous reliable estimates of ARIs and diarrhoea incidence from cohort studies in this area, or in Malawi in general, are available. Estimates of incidence based on LMIC data suggest an impending epidemic of malaria and pneumonia in low and middle-income countries (LMIC) [28, 30] and reports of deficiencies in care raise the concept that young lives are being unnecessarily lost.

Findings in this study suggest the incidence of clinical malaria was 2.5 times (1.2 cases per child-years at risk vs 0.5 cases per child-years at risk) lower than a previous study conducted between 2015 and 2016 in the same area [31]. Before 2016, household ownership of ITNs in the study site was at 29% with a majority having damaged or no ITNs at all [25]. During the current study, a mass ITN distribution campaign, community participation and mobilization, and community-led malaria interventions had been implemented [19, 32]. Studies from Kenya, Rwanda and Uganda have shown the significance of community participation in the formulation of appropriate measures towards malaria control [33, 34]. These interventions may have contributed to the decrease in malaria incidence. However, it should be noted that household ownership of at least one ITN in this community (58.7%) during the time of the study was lower than the 2017 national aggregated estimate (82%) [35].

Despite this disparity, these figures demonstrate a remarkable improvement in ITN ownership at both the community and national levels as evidenced by the previous study [31]. Between 2015 and 2018, the incidence of malaria in Malawi has stagnated between 214 to 217 per 1,000 population at risk despite an increase in the national ITN coverage [30]. Despite this intervention's enormous success, residual malaria transmission cannot be addressed by ITNs or indoor residual spraying (IRS) alone, even at very high coverage [36, 37]. Their long-term viability is jeopardized by a widespread increase in insecticide resistance in the target species [38, 39], which could be the case in the Malawian scenario, where despite an increase in ITN coverage there has been stagnation in clinical malaria incidence. The combined effect of existing interventions with novel strategies involving environmental management such as LSM and socio-economic development through house improvement provides a non-insecticidal, complementary approach to increasing protection against mosquito bites [40, 41]. These supplementary interventions could help to halt malaria transmission by reducing and preventing human-vector contact within residential areas. District-level malaria incidence estimates are however unavailable but could have been better if available for comparison sake.

Malnutrition is one of the possible risk factors for malaria infection among the under-five population in this rural area. At recruitment, some children in the area were discovered to be stunted, underweight and wasted. These conditions increase the children's susceptibility to infection. The presence of anaemia could be another contributing factor. The common causes of anaemia in Malawian under-five children include malaria, deficiencies in iron, folate, and other micronutrients, intestinal worms, and sickle cell disease. Though not specific to malaria as elaborated above, trends in anaemia prevalence can reflect malaria morbidity and have been illustrated in how they respond to changes in the coverage of malaria interventions [42]. Few cases of anaemia were recorded in this study compared to the previous study [31], with a majority being mild anaemia cases, suggesting that the intervention coverage that began in 2016 may have had an impact on malaria, resulting in reduced anaemia cases.

Clinical malaria was unevenly distributed among children within different age-groups and within the focal areas and intervention arms. There were 194 (70.8%) children that did not experience any clinical malaria, 80 children had a total of 110 clinical malaria infections, with some experiencing a repetition of infections. The majority of these cases occurred in focal area B. There are different reasons why focal area B had more malaria cases and repeated infections than other focal areas. Firstly, the Shire River, being the largest river in Malawi, flows through the district, including focal area B. This promotes *Anopheles* mosquito proliferation [43]. Furthermore, due to other agricultural and economic activities being conducted in the area, there is a development of multiple mosquito larval habitats which include cattle hoof prints, rice paddies, brick-pits, and wells [43]. A high number and repeated malaria infections in focal area B suggests the presence of a hotspot with higher malaria transmission than the surrounding areas [44]. A malaria transmission hotspot is defined as a geographical area within a malaria transmission focus where transmission intensity surpasses the average level [44]. Hotspots serve as foci for malaria transmission in most areas, particularly those undergoing malaria elimination, highlighting the importance of establishing targeted control within these areas. In this area, this could be accomplished through targeted interventions to reduce the human infectious reservoir, such as reactive screening and treatment of individuals diagnosed with malaria at health facilities [45], proactive case detection, which involves screening people in hotspots at regular intervals [45], and mass drug administration where a full therapeutic dose of drugs are administered to a population without prior screening. Targeted vector control activities, such as increasing ITN and IRS coverage [46, 47] and larviciding [48] of mosquito breeding sites, can be carried out; however, these interventions are laborious and costly in this context. In this study, 29% of households reported having open eaves, a decline of 10% from the previous study. Closing open eaves in houses has been shown in previous studies to reduce mosquito entry and anaemia in children [9, 49, 50]. A reduction of incidence in this area generally could be attributed to the fact that most houses in the area had closed eaves during the community-led implementation, limiting infectious bites from malaria mosquitoes.

In this study, malaria incidence in southern Malawi was observed peaking during and immediately after the hot-wet season. An increase in the number of mosquito breeding sites during and post rainfall is likely the main contributor to an increase in incidence in this area. A similar pattern of malaria incidence was observed in a previous study in the same area [31]. There are various studies supporting the significance of weather factors in malaria. Studies previously done have shown that temperature and humidity affect mosquito breeding and larval and malaria parasite development [51].

### Incidence of diarrhoea and ARI

The incidence of diarrhoea was low with the highest incidence in children aged 12.0–23.9 months. Although 87% of children had no episodes of acute diarrhoea, 36 children had a total of 47 episodes, with only a few having two or more episodes. In contrast, previous reviews have reported that the highest burden of disease has remained consistent with age for the past 30 years (i.e. 6.0–11.0 month-olds at both global and in the region) [52]. The higher the age the lower the incidence. For instance, in 2010, in the WHO Africa region, the incidence rate among 12.0–23.9 months old was 4.2 episodes/child-years compared to 2.7 episodes/child-years in the 24.0–59.0 months’ age-group. The rise in the incidence of diarrhoea at the age of 12.0–23.9 months can be explained by the possible introduction of contaminated weaning foods by most mothers during this time [53]. There is normally a sequential decrease in the risk after the 6–11 months age category and this is attributed to the development of immunity following repeated exposure to pathogens after this age group [53]. Generally, this correlates with findings in this study which show a decrease in the incidence of diarrhoea with an advance in age.

An important finding from this study is that the ARIs displayed seasonal trends in incidence; ARIs peaked during the cold dry season, suggestive of increased susceptibility to respiratory infections during this season. ARIs may occur more often among under-five children because of their anatomical structure [54, 55]. This age category is still undergoing development of organs such as lungs and is known to have relatively immune immaturity which makes them more vulnerable to infection [54, 55].

This study was not without limitations. There could be rare possibilities that some cases of malaria may have been missed if health care workers did not record a sick visit on a sick-visit card provided or health passport, due to other challenges encountered such as emergencies or a guardian had forgotten the child’s health passport and sick-visit card. Larger sample size and a much longer follow-up period could have been better for the follow-up of these conditions. A period of one year was not adequate for follow-up of these conditions. The other limitation was that the trial was not powered to interpret incidence by trial arm. Furthermore, a generalized linear mixed model to account for within and between cluster effects was not fitted. As a result, the precision of the IRR estimates may be slightly affected (Additional files 2, 3, 4, 5, 6, 7, 8, 9, 10, 11, 12, 13, 14).

## Conclusion

Considering that the current study area is rural, the findings of this study show that the incidence of clinical malaria, ARIs and diarrhoea was low. It was found that, compared to a pre-intervention malaria incidence study, malaria incidence in under-five children had significantly declined. Integrating malaria control strategies as a community participatory initiative has the potential to reduce malaria incidence as it allows a better understanding of the disease and the way it can be controlled, as shown in this community. However, there is a need for community engagement to be as comprehensive as possible by incorporating other major causes of childhood morbidity, such as ARIs and diarrhoea, to improve understanding concerning all diseases. This study highlights the need for more comprehensive studies on malaria, ARIs and diarrhoea to develop more effective interventional strategies to prevent and treat these conditions that impose a significant public health and socio-economic burden in resource-limited countries. Monitoring the epidemiology of these conditions in children may aid in the planning of local service expansion, educational programmes, and preventive measures.

## Supplementary Information

**Additional file 1.** Appendix 1: Map of Majete Wildlife Reserve and the Majete Perimeter.

**Additional file 2.** Appendix 2: Booklet containing diagnosis codes.

**Additional file 3.** Appendix 3: Malaria events.

**Additional file 4. **Appendix 4: Acute respiratory infections (ARIs) events.

**Additional file 5.** Appendix 5: Diarrhoea events.

**Additional file 6.** Appendix 6: MMP focal area A weather data.

**Additional file 7. **Appendix 7: MMP focal area B weather data.

**Additional file 8.** Appendix 8: MMP focal area C weather data.

**Additional file 9.** Appendix 9: Analysis of monthly follow-ups.

**Additional file 10.** Appendix 10: Malaria, ARIs, and diarrhoea events by month.

**Additional file 11.** Appendix 11: Monthly incidence and graphs for ARIs, and diarrhoea.

**Additional file 12.** Appendix 12: Weather and monthly malaria incidence.

**Additional file 13.** Appendix 13: Anthropometry data.

**Additional file 14.** Appendix 14: Data dictionary for the study.

## Data Availability

The dataset is accessible to the corresponding author upon a reasonable request.

## Abbreviations

AL: Artemether-lumefantrine
ARI: Acute respiratory infection
BCC: Behaviour change communication
COMBI: Communication for behavioural-impact
HI: House improvement
IEC: Information, education and communication
IRR: Incidence rate ratio
IRS: Indoor residual spraying
ITN: Insecticide-treated bed nets
LSM: Larval source management
LMIC: Low and middle-income countries
MMP: Majete Malaria Project
RDT: Malaria rapid diagnostic test
MWR: Majete Wildlife Reserve
PCA: Principle component analysis
rMIS: Rolling malaria indicator survey
WHO: World Health Organization
URTI: Upper respiratory tract infection
