# Supplementary material for: Incidence of clinical malaria, acute respiratory illness, and diarrhoea in children in southern Malawi: a prospective cohort study
Source: Malar J. 2021 Dec 20;20:473. doi: 10.1186/s12936-021-04013-5 (PMC8685799; doi:10.1186/s12936-021-04013-5)

**Additional File 1**

Appendix 1: Map of Majete Wildlife Reserve and the Majete Perimeter showing the three focal areas. Adapted with permission from Kabaghe et al [21].


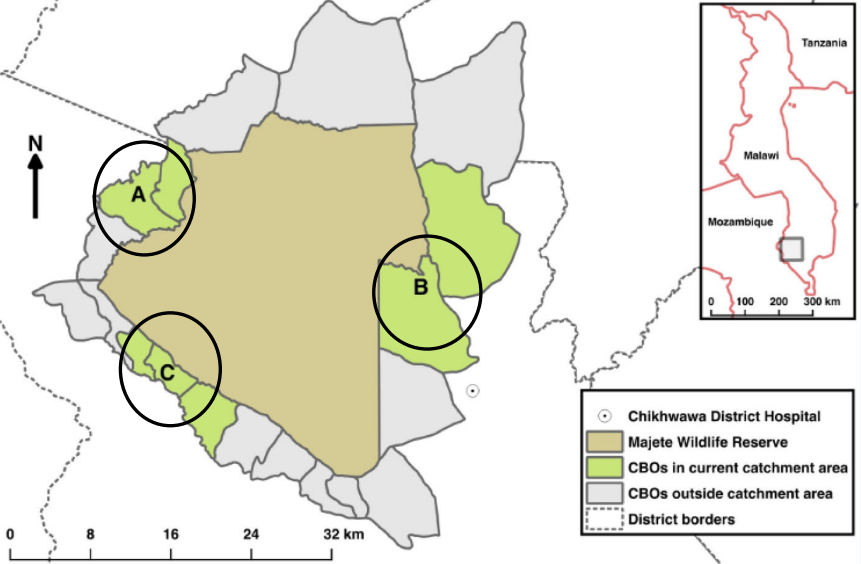

Supplement: Supplementary file 1 — Additional file 1. Appendix 1: Map of Majete Wildlife Reserve and the Majete Perimeter. [file 12936_2021_4013_MOESM1_ESM.docx]
