# Supplementary material for: Incidence of clinical malaria, acute respiratory illness, and diarrhoea in children in southern Malawi: a prospective cohort study
Source: Malar J. 2021 Dec 20;20:473. doi: 10.1186/s12936-021-04013-5 (PMC8685799; doi:10.1186/s12936-021-04013-5)
Supplement: Supplementary file 2 — Additional file 2. Appendix 2: Booklet containing diagnosis codes. [file 12936_2021_4013_MOESM2_ESM.pdf]

BOOKLET CONTAINING DIAGNOSIS CODES

INCIDENCE STUDY (UNDER-FIVE)

No	W.H.O CODE	ACUTE DIARRHEA
D1	190352	**NO PATHOGEN CONFIRMED (PROBABLE)**<br>**All of the following:**<br>1. Clinical syndrome with acute onset of three or more unformed bowel movements (or stools) in a 24-hour period<br>*and*<br>2. Duration lasting greater than or equal to three days and less than or equal to 14 days<br>*and*<br>3. No pathogen identified
		**CHRONIC DIARRHEA**
D2	190362	**NO PATHOGEN CONFIRMED (PROBABLE)**<br>**All of the following:**<br>1. Clinical syndrome of three or more unformed bowel movements (or stools) in a 24-hour<br>period<br>*and*<br>2. Duration greater than or equal to (≥) 28 days if greater than or equal to (≥) 15 years of age, and greater than or equal to (≥) 14 days if age less than (<) 15 years<br>*and*<br>3. Either diagnostic testing was done and no pathogen was identified or diagnostic testing was not available
		**COUGH**
C1	190652	**Influenza**<br>**NO PATHOGEN CONFIRMED (PROBABLE)**<br>**Both of the following:**<br>1. Influenza-like illness as defined by the sudden onset of fever and cough or sore throat in the absence of other diagnoses<br>*and*<br>2. Evidence of regional endemic/epidemic area or travel to endemic/epidemic area
C2	191042	**PNEUMONIA, BACTERIAL SINGLE EPISODE** within six months<br>**NO PATHOGEN CONFIRMED (PROBABLE), single episode within six months**<br>All of the following in a patient with no previous episode of pneumonia in past six months:<br>1. At least one of the following:<br>a. Fever or cough, Shortness of breath, Chest pains<br>or<br>b. New onset of purulent sputum or change in character of sputum<br>and

		2. Appropriate antibacterial therapy initiated or recommended
**C3**	**191062**	**PNEUMONIA, NON-BACTERIAL**<br>**NO PATHOGEN CONFIRMED (PROBABLE)**<br>All of the following:<br>1. Compatible clinical syndrome of pneumonia (e.g. productive cough and fever)<br>and<br>2. Radiologic evidence of pulmonary infiltrate<br>and<br>3. Antibacterial therapy not initiated
**C4**	**220022**	**MYCOBACTERIUM TUBERCULOSIS PULMONARY**<br>**NO PATHOGEN CONFIRMED (PROBABLE)**<br>**AGE < 2 YEARS**<br>All of the following:<br>1. At least one of the following:<br>a. Non-specific signs and symptoms are present, such as productive or non-productive chronic cough, hemoptysis, fever, night sweats, or anorexia or weight loss (child with weight less than (<) 3rd percentile for age or a decrease in weight that has crossed two major growth percentiles since the last documented weight)<br>or<br>b. Abnormal chest x-ray (e.g., hilar adenopathy, paratracheal lymphadenopathy, alveolar consolidation, miliary pattern, parenchymal breakdown/cavitation, or Ghon focus).<br>and<br>2. History of contact with an individual with known or suspected tuberculosis (TB) and<br>3. At least one of the following:<br>a. No response achieved with standard broad spectrum antibiotic treatment<br>or<br>b. Specific anti-tuberculous therapy initiated<br><br>**AGE > 2 YEARS**<br>All of the following:<br>1. Compatible clinical symptoms (e.g., cough, hemoptysis, shortness of breath, chest pain, weight loss, fever, or night sweats of greater than or equal to (≥) two weeks duration)<br>and<br>2. At least one of the following:<br>a. Positive sputum smear for acid fast bacilli (AFB)<br>or<br>b. Either abnormal chest x-ray, chest CT scan or other chest imaging (e.g., hilar lymphadenopathy, paratracheal lymphadenopathy, alveolar consolidation, miliary pattern, parenchymal breakdown/cavitation, or Ghon focus)<br>or

		c. Evidence of granulomata with organisms positive for AFB or caseating granulomata on lung tissue biopsy or at autopsy<br>or<br>d. Positive tuberculin skin test (TST) (> five mm) or interferon gamma release assay (IGRA)<br>and<br>3. Without concurrent illness that would explain the findings
		**ASTHMA, CHRONIC**
**C5**	**300091**	**CONFIRMED**<br>Both of the following:<br>1. Two or more recurring episodes of sudden onset of wheezing and cough; may or may not be associated with a concurrent infection, drug exposure or exposure to another antigen<br>and<br>2. Episode is self-limited; may respond to treatment
		**BRONCHIOLITIS**
**C6**	**190092**	Clinical syndrome of wheezing, cough, and hypoxia typically seen in children less than (<) two years of age, most often caused by inflammation of the bronchioles and lower respiratory tract, due to infection with respiratory syncytial virus (RSV) or, on occasion, parainfluenza virus, influenza virus,<br>or adenovirus<br>**NO PATHOGEN CONFIRMED (PROBABLE)**<br>**Both of the following:**<br>1. Clinical diagnosis made by experienced practitioner<br>*and*<br>2. Microbiologic tests negative or not done
**C7**	**190292**	**CROUP (LARYNGOTRACHEOBRONCHITIS)**<br>Respiratory disease characterized by a barking cough typically affecting infants and children aged three month to three years. The syndrome includes sudden onset of respiratory stridor with upper respiratory infection; inflammation of larynx, trachea, or bronchi is seen with severe illness. The most common etiology is parainfluenza virus, usually type 1 or 3; however, other viruses and bacteria may be the causative agent.<br>**NO PATHOGEN CONFIRMED (PROBABLE)**<br>**Both of the following:**<br>1. Clinical syndrome<br>and<br>2. Negative culture or rapid diagnostic tests or tests not done
**R1**	**190782**	**MEASLES - also called RUBEOLA**<br>Highly-contagious viral disease that causes fever, cough, catarrh, and conjunctivitis followed by characteristic rash; complications include pneumonia, encephalitis, and death<br>**NO PATHOGEN CONFIRMED (PROBABLE)**<br>**Both of the following**:<br>1. Appropriate clinical syndrome<br>and<br>2. Virus-specific serology or culture not done

**R2**	**191262**	**VARICELLA-ZOSTER VIRUS (VZV) – CHICKEN POX**<br><br>Specify zoster, herpes zoster, or shingles and disseminated in the description<br>**NO PATHOGEN CONFIRMED (PROBABLE)**<br>**All of the following**:<br>1. Appropriate clinical diagnosis of rash typical of herpes zoster or shingles, first episode<br>*and*<br>2. Without complications<br>*and*<br>3. Diagnostic culture, nucleic acid amplification test, or antigen tests negative or not done
**R3**	**130041**	**ATOPIC DERMATITIS/ECZEMA**<br>**CONFIRMED**<br>Dermatitis of unknown etiology characterized by itching and scratching in an individual with inherently irritable skin
**R4**	**130231**	**SCABIES**<br>**CONFIRMED**<br>Superficial infection causing a pruritic rash typically occurring in skin folds and exacerbated by heat or hot water; ectoparasites documented by microscopy
**R5**	**190342**	**DERMATOPHYTE INFECTIONS (TINEA or RINGWORM)**<br>**NO PATHOGEN CONFIRMED (PROBABLE)**<br>1. Infection suspected clinically<br>*and*<br>2. Specific tests negative or not done
